# OIE Annual Report on Antimicrobial Agents Intended for Use in Animals: Methods Used

**DOI:** 10.3389/fvets.2019.00317

**Published:** 2019-09-25

**Authors:** Delfy Góchez, Margot Raicek, Jorge Pinto Ferreira, Morgan Jeannin, Gerard Moulin, Elisabeth Erlacher-Vindel

**Affiliations:** ^1^Antimicrobial Resistance and Veterinary Products Department, World Organisation for Animal Health (OIE), Paris, France; ^2^Agence nationale de Sécurité Sanitaire, Alimentation, Environnement, Travail (ANSES), Fougères, France

**Keywords:** antimicrobial resistance (AMR), antimicrobial use (AMU), report, methods/methodology, surveillance, monitoring

## Abstract

For over two decades, the World Organisation for Animal Health (OIE) has engaged in combatting antimicrobial resistance (AMR) through a One Health approach. Monitoring of antimicrobial use (AMU) is an important source of information that together with surveillance of AMR can be used for the assessment and management of risks related to AMR. In the framework of the Global Action Plan on AMR, the OIE has built a global database on antimicrobial agents intended for use in animals, supported by the Tripartite (World Health Organization (WHO), Food and Agriculture Organization of the United Nations (FAO) and OIE) collaboration. The OIE launched its first annual data collection in 2015 and published the Report in 2016. The second Report, published in 2017, introduced a new methodology to report quantitative data in the context of relevant animal populations, and included for the first time an annual analysis of antimicrobial quantities adjusted for animal biomass on a global and regional level. A continuing annual increase of countries participating in the data collection demonstrates the countries engagement for the global development of monitoring and surveillance systems in line with OIE international standards. Where countries are not yet able to contribute their quantitative data, their reports also highlight the barriers that impede them in data collection, analysis and/or reporting. The OIE Reports show annual global and regional estimates of antimicrobial agents intended for use in animals adjusted for animal biomass, as represented by the quantitative data reported by countries to the OIE. The OIE advises caution in interpretation of estimates made in the first few years of reporting recognizing some important limitations faced by countries as they develop their monitoring systems. The OIE remains strongly committed to supporting its Members in developing robust and transparent measurement and reporting mechanisms for AMU.

## Introduction

The World Organisation for Animal Health (OIE) has worked actively for more than two decades on veterinary products, including antimicrobial agents, and developed a coherent strategy for its activities in this area (1). Monitoring of antimicrobial use (AMU) is an important source of information that, together with surveillance of AMR, can be used for the assessment and management of risks related to AMR. Toward standardization of surveillance and monitoring data worldwide, the OIE developed standards on “Monitoring of the quantities and usage patterns of antimicrobial agents used in food producing animals” [(2) Terrestrial Animal Health Code Chapter 6.9.], “Monitoring of the quantities and usage patterns of antimicrobial agents used in aquatic animals” [(3) Aquatic Animal Health Code Chapter 6.3.] and on the “Harmonization of national antimicrobial resistance surveillance and monitoring programmes” [(2) Terrestrial Animal Health Code Chapter 6.8.], and “Development and harmonization of national antimicrobial resistance surveillance and monitoring programmes for aquatic animals” [(3) Aquatic Animal Health Code Chapter 6.4] (3).

In the framework of the Global Action Plan on AMR, the OIE has also built a global database on antimicrobial agents intended for use in animals, supported by the Tripartite collaboration (WHO, FAO, OIE).

The OIE *ad hoc* Group on Antimicrobial Resistance developed a template for harmonized AMU data collection, as well as guidance for its completion that are available in the three official OIE languages (i.e., English, French, and Spanish) (4).

The OIE launched its annual data collection on AMU in 2015, and published the first Report in 2016 (5).

The second Report, published in 2017, introduced a new methodology to report quantitative data in the context of relevant animal populations and included for the first time an annual analysis of antimicrobial quantities adjusted for animal biomass on a global and regional level (6). The third report using the same methodology was published in February 2019 (7). The OIE animal biomass methodology was developed with the goal of best representing animal biomass in all OIE Regions, with different animal populations and production systems, and data collection systems, using the data available at the international level.

The methodology for the animal biomass calculation was developed with the support and validation of the OIE *ad hoc* Group on Antimicrobial Resistance, and shared with Member Countries in the report of the OIE Scientific Commission for Animal Diseases meeting of September 2017.

The methodology for calculating animal biomass on a global level, used by the OIE for analysis of reported data on antimicrobial agents intended for use in animals, is detailed in this article.

## Materials and Methods

### OIE AMU Data Collection

Each year in October, the template and accompanying guidance documents are sent to all 182 OIE Member Countries and 11 non-OIE Member Countries. The deadline for submission is December, but responses may be accepted until mid-May of the following year.

The template, to be completed by the respondents, is provided in the form of an Excel file that includes four worksheets labeled “Baseline Information,” “Reporting Option 1,” “Reporting Option 2,” and “Reporting Option 3.”

The “Baseline Information” sheet can be answered by any country, and collects general information on topics including the use of antimicrobials as growth promoters, and any barriers to reporting quantitative data on antimicrobial agents used in animals.

For countries able to provide quantitative data on antimicrobial agents intended for use in animals, the Baseline Information sheet also contains questions relevant to data collection such as data sources, year and animal species covered by the reported data.

Following completion of the Baseline Information, the template either directs countries to submit the questionnaire if no quantitative data are available, or to complete one of the three “Reporting Options” if quantitative data are available. The three reporting options represent increasing levels of detail of quantitative data on antimicrobial classes used in animals, with the possibility of separating amounts reported by type of use (“veterinary medical use,” which includes use to treat, control, or prevent disease; and “non-veterinary medical use,” which includes use for growth promotion), animal groups (terrestrial food-producing, aquatic food-producing, or companion) and routes of administration.

#### Antimicrobial Agents Reported

For the harmonization of the submitted data, the OIE established the List of antimicrobial classes to be reported by the participant countries (Table 1). Data on antimicrobials sold/imported/prescribed/used in the country in animals are reported at the class/subclass level. All pharmaceutical forms are included. The quantities for each antimicrobial class can be reported either for veterinary medical use (including prevention of clinical signs) or for growth promotion purpose.

**Table 1:** Classes of antimicrobial agents for reporting.

**Antimicrobial class**	**Guidance**
Aminoglycosides	Includes aminocyclitols (e.g., streptomycin, dihydrostreptomycin, and spectinomycin) and all other aminoglycosides (e.g., gentamicin, kanamycin, neomycin, apramycin).
Amphenicols	Includes florfenicol and thiamphenicol.
Arsenicals	Includes nitarsone, roxarsone, and others.
Cephalosporins	May be reported as, • Cephalosporins (all generations) or • In relevant category groupings: ∘ 1–2 generation cephalosporins and ∘ 3–4 generation cephalosporins
Fluoroquinolones	Includes danofloxacin, difloxacin, enrofloxacin, marbofloxacin, and other fluoroquinolones, but not other quinolones (e.g., flumequine, oxolinic acid, nalidixic acid), which are reported separately.
Glycopeptides	Includes avoparcin and others.
Glycophospholipids	Includes bambermycin (i.e., flavomycin).
Lincosamides	Includes lincomycin, pirlimycin, and others.
Macrolides	Includes substances with all macrolide structures, such as erythromycin, spiramycin, tylosin, tylvalosin, gamithromycin, tildipirosin, tulathromycin, and others.
Nitrofurans	Includes furazolidone, nitrofurantoin, nitrofurazone, and others.
Orthosomycins	Includes avilamycin and others.
Other quinolones	Includes flumequine, nalidixic acid, oxolinic acid, and others.
Penicillins	Includes all penicillins (e.g., natural penicillins, aminopenicillins, and others), but excludes other beta lactam antimicrobials like cephalosporins.
Pleuromutilins	Includes tiamulin, valnemulin, and others.
Quinoxalines	Includes carbadox, olaquindox, and others.
Streptogramins	Includes virginiamycin, pristinamycin, and others.
Sulfonamides (including trimethoprim)	Includes all sulfonamides, as well as trimethoprim, and similar compounds.
Tetracyclines	Includes chlortetracycline, doxycycline, tetracycline, and oxytetracycline.
Others	All others not covered, including coumarin antimicrobials, e.g., novobiocin, fusidic acid, kirromycins, phosphonic acids like fosfomycin, rifamycins, thiostrepton.

If there are confidentiality or proprietary reason that imped a country to individually report the quantities for one or more antimicrobial classes, such quantities should report as “Aggregated class data,” an existing category in all three Reporting Options proposed by the OIE. The country that uses this category should list the names of the antimicrobial classes that cannot be reported individually.

For each cycle of data collection, a specific year is targeted—for example, data from 2015 for the third report published in 2019. However, countries with more recent quantitative data may also report that data.

For each reported year, the country informs the OIE on the period of time covered within the year, the data sources (Table 2), coverage of the data (if is <100% the country explains which quantitative data is inaccessible), animal groups covered (terrestrial food-producing animals, aquatic food-producing animals, and companion animals), food-producing species covered, species considered companion animals covered by the data and the link to the national report available on the web, if any.

**Table 2:** Data Sources proposed by the OIE.

Sales data	Wholesalers
	Retailers
	Marketing authorization holders
	Registration authorities
	Feed mills
	Pharmacies
	Farms shops/agricultural suppliers
	Industry trade associations
Purchase Data	Wholesalers
	Retailers
	Feed mills
	Pharmacies
	Agricultural cooperatives
	Producer organizations
Import data	Customs declarations—veterinary medicinal product
	Customs declarations—active ingredients
Veterinary data	Sales
	Prescriptions
Antimicrobial use data	Farm records
Other data source(S)	Other—free text field

#### Calculation of Kilograms of Active Ingredients

For the purpose of reporting data on antimicrobial quantities (amounts sold or imported for use in animals expressed in kilograms (kg) of antimicrobial agent, i.e., chemical compound as declared on the product label, that is to be calculated from the available information), animals are grouped into “all animal species,” “companion animals,” “all food-producing animals,” “terrestrial food-producing animals,” and “aquatic food-producing animals.”

The amount of the antimicrobial agents intended for use in animals in kilograms (kg) (the chemical compound or active ingredient as declared on the product label, that is to be calculated from the available information) should be reported. Where data are available in the form of number of packages of a given pharmaceutical preparation sold/imported/prescribed/used; international units (IU) and; percentage weight per volume (% w/v), mathematical conversion are necessary in order to report the kilograms of active ingredients to the OIE.

Ideally, the OIE is interested in the amount of active ingredient (moiety), that is, the substance as listed in the *OIE List of antimicrobial agents of veterinary importance* (8) (e.g., benzylpenicillin), not the total weight of the actual chemical compound (salt, ester, or other, for example: sodium or potassium benzylpenicillin) contained in a veterinary medicinal product or traded as bulk material. At this stage of the project, the precision gained by the refined reporting of amounts of active ingredient, achieved by mathematical conversion of amounts of chemical compound as declared on the product label, is not justified. Therefore, the OIE template accepts the amounts of chemical compound as declared on the product label.

Since the second year of data collection, a question was added to the template in order to understand the barriers impeding countries from reporting amounts of antimicrobial agents in animals. This information is useful for guiding discussion on overcoming barriers during training Seminars of National Focal Points for Veterinary Products (those who most frequently complete the OIE Template) and increasing availability of quantitative data in the future, and reflects challenges in National Action Plan implementation that would also be assessed during the Performance of the Veterinary Services (PVS pathway) evaluation.

The barriers highlighted by responding countries are grouped into four main categories (Lack of regulatory framework, Lack of coordination/cooperation between national authorities and with private sector, Lack of tools and human resources, and Insufficient regulatory enforcement).

#### Validation of the Data and Calculations Performed at the OIE Level on Antimicrobials Quantities

The OIE systematically reviews the reported information and systematically comes back to responding countries to clarify some issues, or to request missing data.

Whenever possible, the data reported by countries are checked by the OIE against existing reference sources, using the previous year's reported data and/or national reports available online. The indicator for this comparison is a calculated “percentage of change” relative to the reference.

In the countries with high percentages of unexplained change (> ±25%), the OIE inquires how the calculations to obtain kg of antimicrobial agents sold/used were carried out. Through this process, errors in the calculations can be identified and corrected.

When all the responses have been validated, the OIE proceeds with analysis toward preparation of the report. The amounts of antimicrobials sold/used are calculated by country, by region, by animal groups, type of use, and pharmaceutical forms.

As all the countries do not provide the same level of details, some calculations are done on only a part of the countries providing quantitative data.

Through this process, the final result is a quantity of antimicrobials sold/used expressed in kg/country/year. As many countries are still in the process of developing their data collection systems, these results are reported by the OIE on the global and regional level at this stage.

### OIE Calculation of the Animal Biomass

To compare quantitative data reported on antimicrobial agents intended for use in animals between regions and over time, a scale is necessary to evaluate these data in the context of the relevant animal populations, which vary in size, and composition.

Therefore, the quantitative data reported on antimicrobial agents intended for use in animals needs to be adjusted for the relevant animal biomass according to the following calculation:

$$\begin{array}{l} \frac{antimicrobial\;agents\;reported\;\left(mg\right)}{animal\;biomass\;\left(kg\right)\;} \end{array}$$

Animal biomass is calculated as the total weight of the live domestic animals in a given population present during a year in a specific area, used as a proxy to represent those likely exposed to the quantities of antimicrobial agents reported.

Animal biomass is currently employed as a denominator in analysis of quantitative antimicrobial use data by other national and regional antimicrobial use surveillance groups, such as the European Surveillance of Veterinary Antimicrobial Consumption (ESVAC)—(9), the Canadian Integrated Program for Antimicrobial Resistance Surveillance (CIPARS)—(10), and the Japanese Veterinary Antimicrobial Resistance Monitoring System (JVARM, (11)). In 2017 the US FDA proposed a method for estimating a Biomass denominator (12).

While several methodologies have been developed for the calculation of animal biomass by other surveillance groups, none could be directly used for the OIE global database on antimicrobial agents intended for use in animals. Particularly, these methodologies utilize available data on animal populations detailed by production class, estimates of live animal weights, import/export data, and total annual populations of production groups living <1 year (i.e., poultry, veal calves, fattening pigs, lambs, and kids). On a global level, such detailed data are not yet available for many countries, and therefore a new methodology was developed by the OIE mainly using globally available datasets—the OIE World Animal Health Information System (WAHIS) and the United Nations Food and Agriculture Organization Statistics (FAOSTAT).

The formulas for calculating biomass by species were developed using the two globally available datasets, WAHIS and FAOSTAT, and were compared to references from countries where more detailed animal population data by production class were available. These references include animal biomass figures either directly supplied from Member Countries, or calculated from animal population data in Eurostat, the statistical office of the European Union.

The formulas chosen for calculation of the OIE denominator reflect the best fit estimations using the more general global animal population data (WAHIS, FAOSTAT) when compared to these available reference figures. The derived formulas were then applied to all countries providing quantitative data for the target year.

All weights and biomass figures are measured in kilograms (kg).

Data collected by these global animal surveillance databases, WAHIS and FAOSTAT, are point in time species-level census data^1^ with little to no detail on the production class. Such data are difficult to interpret given that production classes within a species can have very different average weights, such as beef cattle and veal calves. Additionally, given that census data are collected at one point in time of the year, the total annual population is not known for production groups which are slaughtered and repopulated a certain number of times within 1 year (this multiplication factor is hereafter referred to as “cycle factor”).

In development of the methodology for calculation of an annual animal biomass, the underlying effort was to best use globally available census data from the OIE WAHIS interface. WAHIS data are reported by National Veterinary Services through OIE Focal Points for Animal Disease Notification, and the figures are subsequently validated by OIE staff.

FAOSTAT animal population data are used as a complementary dataset. FAOSTAT data are similarly primarily obtained from national governments, but sources expand beyond National Veterinary Services to National Statistics Offices and other relevant agencies. When a national government does not report a figure to FAOSTAT, FAO uses local expert resources to estimate a figure, or their statistical team to imputate a data point. The two datasets are therefore similar but can display significant variation.

Where census data were used, the WAHIS and FAOSTAT figures are first cross-referenced with each other, and then with national reports or literature as needed. FAOSTAT data are used when a WAHIS data point is not available or is outside of expected variation without explanation.

In addition to census data, FAOSTAT also reports numbers and tons of production animal species slaughtered by country each year, similarly undifferentiated by production class. As WAHIS does not collect this information, FAOSTAT slaughter data is used when these data were needed. For species living <1 year, it was necessary to use data on number of animals slaughtered to represent an annual population, as this information cannot be extrapolated from point in time census data without a cycle factor specific to each country's production model.

## Results

### Principles of Animal Biomass Calculation Methodology

The overall objective of the methodology is to obtain the biomass of animals present during the year of analysis in a specific country using internationally available data.

For a given species, animal weight varies by age and production class, and therefore the structure of the population of a given species must be taken into account.

The first approach is to distinguish animals from production classes with a lifespan >1 year, and those with a lifespan of <1 year.

For animals living for more than 1 year, it was considered that census data (number of animals present at one time) can be a good basis to evaluate the number of animals present in the country during the year.

In this case, the biomass can be obtained by multiplying the number of animals present at one point in time (census data) by a calculated weight (if available or possible to calculate) or standard weight.

Generally, census data available represent the number of animals present at one point in time and include all animals within the species regardless of their age and production class. Thus, it is necessary to estimate the number of adults vs. young animals to ensure appropriate average weights were applied. Different methods for this estimation were used depending on the species and available data.

For example, to differentiate breeding swine (sows) with a production lifespan >1 year, from the fattening pigs, an estimation of the percentage of sows in the total pig population was calculated based on Eurostat data, where production-class detailed information is available. To calculate the number of sows, the percentage obtained was applied to the census data as a constant for all the countries. For cattle, the approach is different due to the large variation of body weight between production classes (calves, young cattle, and adults), and a broader diversity of production models (veal meat, beef meat, milking cows, etc…). For this species a model was built to estimate the structure of an average bovine population, based on Eurostat production-class information, and was applied to all the countries.

The mean weight of adult animals was generally based on existing standard weights, adapted regionally by livestock unit classification (13). For animals living <1 year, production data (number and weight of animals slaughtered annually) were considered as a good basis to estimate the average weight of animals present in the country during the year.

The application of these principles for calculating mean weights depends on the species; in some species, like poultry, mainly young animals are slaughtered (with a production life less than a year). In other species like cattle, goats, sheep, and swine, despite a large number of young animals being slaughtered, adults (with a production life greater than a year) may represent a significant portion of the population.

### Calculation Methodology of Average Animal Weights

Different antimicrobial use surveillance programmes have used various methodologies for determining the average animal weights used in the calculation of total biomass.

In the European Surveillance of Veterinary Antimicrobial Consumption (ESVAC), estimated average weights at time of treatment are used (9). The Canadian Integrated Surveillance Program for Antimicrobial Resistance (CIPARS) also uses the same estimated weights at time of treatment, as well as Canadian standard weights (10). The surveillance programs of Japan and the United States (12) take a different approach, instead using more general estimates of average animal weights by production category, rather than focusing the estimates on an average size at treatment.

On a global scale, it was not considered feasible to estimate weights at time of treatment for all countries reporting data to the OIE. The live weight of the animal before slaughter was most easily accessible, and was considered to be a best representation of average weights for a global calculation of animal biomass.

The live weights of animals before slaughter were calculated using FAOSTAT annual slaughter data, for all species and regions where these data were available, using the following two formulas:

$$\begin{array}{l} carcass\;weight\;\left(kg\right)=\;\frac{total\;weight\;of\;species\;slaughtered\;\left(kg\right)}{number\;of\;species\;slaughtered\;\left(heads\right)} \end{array}$$

Carcass weights were converted to live weights at time of slaughter using conversion coefficients (k) as defined by Eurostat, also known as dressing percentages (14). Conversion coefficients represent the difference between a processed carcass weight and the expected live weight of that animal species before slaughter, expressed as a fraction.

$$\begin{array}{l} live\;weight\;\left(kg\right)=\;\frac{carcass\;weight\;\left(kg\right)}{conversion\;coefficient\;\left(k\right)} \end{array}$$

**Bovine (including cattle and domestic buffalo)** biomass was calculated according to the following principles:

1. Countries were grouped by sub-region as defined by livestock unit classifications (13). A sub-regional mean live weight was then determined by calculating the average live weight of bovines for countries within the sub-regional grouping from their production data;
2. From the calculated sub-regional mean live weight, the weights of the different bovine production categories [adults, young (between 1 and 2 years of age), calves (<1 year of age)] were determined by applying relevant weight proportions standards, originating from livestock unit ratios defined by Eurostat (15). Consecutively, the weight of each bovine production category was then multiplied by a predicted population ratio. These population ratios were calculated in the reference Eurostat database and consider an anticipated renewal rate of 30%.

**Bovine** biomass was calculated by multiplying the representative weight determined for each sub-region by the census population of bovines for each country within the sub-region, according to the following formula:

$$\begin{array}{l} census\;population\times [(sub\;regional\;mean\;live\;weight\\ \times LSU_{calves}\times P.pop_{calves})+(sub\;regional\;mean\;live\;weight\\ \times LSU_{young\;1-2yrs}\times P.pop_{young\;1-2yrs})\;\\ +\left(sub\;regional\;mean\;live\;weight\times LSU_{adults}\times P.pop_{adults}\right)]\; \end{array}$$

Whereby,

*P.pop*_*calves*_, *P.pop*_*young*_ _1−2*years*_, *P.pop*_*adults*_ represents, respectively, the proportion (P.pop) of calves, young (between 1 and 2 years of age) and adults in the total living cattle population, as calculated from Eurostat animal population data.

LSU_*calves*_, LSU _*young*_ _1−2*years*_, LSU _*adults*_ represents, respectively, the livestock unit ratios (LSU) for calves, young and adults as defined by Eurostat (15).

And, *sub regional mean live weight* represents the calculated mean live weight for adult cattle at the sub regional level.

#### Determination of the Mean Live Weight of Adult Cattle

The mean live weight of adult cattle is estimated by calculating a Generic mean live weight at slaughter from the production data which is then multiplied by a correction factor, derived from the Eurostat reference dataset.

Mean weight of live adult cattle = Generic mean live weight at slaughter ^*^ Correction factor (1.15).

#### Generic Mean Live Weight at Slaughter

The Generic mean live weight at slaughter, comprised of the weights of all the cattle slaughtered regardless of their production category, is calculated from annual production data (FAOSTAT), using the carcass to live weight conversion coefficient (÷ 0.54, formula 4.2.2.2), as defined by Eurostat:

Generic mean live weight at slaughter (kg) = $\frac{Generic\;carcass\;weight\;\left(kg\right)}{\;conversion\;coefficient\;\left(0.54\right)}$

#### Determination of the Correction Coefficient

Using reference datasets (Eurostat and several national detailed reports), where slaughter data are detailed by production category [adults, young (between 1 and 2 years of age), calves (<1 year of age)], it was estimated that, on average, the mean weight of live adult cattle was 15% higher than the Generic mean live weight at slaughter.

$$\begin{array}{l} \frac{mean\;live\;weight\;of\;adults\;at\;time\;of\;slaughter}{Generic\;mean\;live\;weight\;at\;slaughter}=1.15 \end{array}$$

Therefore, applying an add-on factor of 15% (× 1.15) to the Generic mean live weight at slaughter is the best fit model to obtain the mean live weight of adult cattle when compared to the reference datasets (Eurostat and country specific data).

#### Sub-Regional Mean Live Weight

Countries were grouped by sub-region as defined by livestock unit classifications (13). A sub-regional mean live weight was then determined by calculating the average of the mean live weight of adult cattle for countries within the sub-regional grouping.

**Swine** biomass was calculated according to the following formula:

$$\begin{array}{l} \left(live\;weight\;\times \;number\;slaughtered\right)+(census\;population\;\\ \times \;sow\;weight\;\times \;0.09) \end{array}$$

Whereby,

*live weight* × *number slaughtered* represents the expected biomass of fattening pigs slaughtered in a country in 1 year,

And *census population* × *sow weight* × 0.09 represents the expected biomass of pigs retained for breeding purposes, calculated with the following considerations:

◦ The number of boars for breeding purposes is negligible compared to the number of sows;
◦ Sow weight: the standard weight of a sow in Europe is 240 kg (9). This weight was adapted by region using livestock unit ratios (Americas = 240 kg, Asia and the Pacific = 240 kg, Africa = 192 kg);
◦ 0.09 is the expected percentage of sows in a given swine population, as calculated from Eurostat animal population data.

**Poultry** biomass was calculated according to the following formula:

$$\begin{array}{l} \left(live\;weight\;chicken\;\times \;number\;of\;chicken\;slaughtered\right)\\ +\left(live\;weight\;turkey\;\times \;number\;of\;turkey\;slaughtered\right)\\ +\left(live\;weight\;ducks\;\times \;number\;of\;ducks\;slaughtered\right)\\ +\left(live\;weight\;geese\;\times \;number\;of\;geese\;slaughtered\right) \end{array}$$

**Equidae** biomass was calculated according to the following formula:

$$\begin{array}{l} \left(live\;weight\;horse\;\times \;horse\;census\;population\right)\\ +\;\left(live\;weight\;donkey\;\times \;donkey\;census\;population\right)\\ +\;\left(live\;weight\;mules\;\times \;mule\;census\;population\right) \end{array}$$

The live weight of horses, donkeys, and mules was calculated for regions where equine slaughter is common and data were available. For regions where equine slaughter is not practiced and/or where data were unavailable, live weights were adapted using livestock unit ratios.

**Sheep and goat** biomass were calculated according to the following formula:

$$\begin{array}{l} \left(live\;weight\;\times \;number\;slaughtered\right)\;\\ +\left(census\;population-\;\frac{number\;slaughtered}{1.5}\right)\times \;75\;kg \end{array}$$

Whereby,

(*live weight* × *number slaughtered*) represents the expected biomass of sheep and goats slaughtered in a country in 1 year,

And $\left(census\;population-\;\frac{number\;slaughtered}{1.5}\right)\times \;75\;kg$ represents the expected biomass of animals retained for breeding purposes, calculated with the following considerations:

◦ 1.5 is the average number of breeding cycles per year;
◦ The standard weight of a breeding small ruminant in Europe is 75 kg (9). This weight was used globally based on livestock unit ratios.

**Rabbit** biomass was calculated according to the following formula:

$$\begin{array}{l} \left(live\;weight\;\times \;number\;slaughtered\right)\;\\ +\left(census\;population-\;\frac{number\;slaughtered}{5}\right)\times \;4.5\;kg \end{array}$$

Whereby,

(*live weight* × *number slaughtered*) represents the expected biomass of rabbits slaughtered in a country in 1 year,

And $\left(census\;population-\;\frac{number\;slaughtered}{5}\right)\times \;4.5\;kg$ represents the expected biomass of animals retained for breeding purposes, calculated with the following considerations:

◦ 5 is the average number of breeding cycles per year;
◦ The standard weight of a breeding doe is 4.5 kg.

**Camelid and cervid** biomass were calculated according to the following formula:

$$\begin{array}{l} standard\;weight\;\times \;census\;population \end{array}$$

According to the following considerations (16):

◦ Standard weight cervid: 80 kg
◦ Standard weight camel: 600 kg
◦ Standard weight, llama/alpaca: 100 kg

**Farmed fish** biomass was included in the total biomass only for countries that included aquaculture in their reported data on antimicrobials intended for use in animals. Aquaculture data are collected in WAHIS and FAOSTAT as tons produced annually, which were converted to kilograms for the animal biomass calculation.

Data on farmed crustaceans, molluscs and amphibians were excluded given the relatively small size of these populations, and inconsistency in their reporting.

**Cats and dogs** have not yet been included in the calculation of animal biomass due to inconsistency in reporting of their populations, and lack of information on average weights. For the countries where companion animal data was available, their contribution to overall animal biomass was found to be relatively minor (<1%). In the future, an analysis of companion animal data will hopefully become feasible.

## Discussion

### Limitations

The OIE data collection on antimicrobials intended for use in animals is at an early stage of development and caution should therefore be taken when interpreting the data of the first years of data collection.

Multiple data sources are used by the different countries including imports, wholesalers data, marketing authorization holder declarations, and veterinary prescription. The level of accuracy of the data may be different according to the data source, for example import data may be relatively imprecise compared to marketing holder declarations or veterinary prescriptions.

In some cases, reporting of many different data sources can also result in over-estimated, duplicated or overlapping data. The OIE works with its Member Countries to correct thee issues wherever possible.

The OIE will continue to support its Member Countries through its Regional Trainings for National Focal Points for Veterinary Products, where the guidelines are reviewed and Member Countries can ask the OIE questions and share experiences with their peers.

As stated in the annual European Surveillance of Veterinary Antimicrobial Consumption (ESVAC) report, 3–4 years are needed to establish a valid baseline for the data on sales of veterinary antimicrobial agents.

The animal biomass methodology was developed taking into account internationally available data. The level of detail of information available on a global level necessitated the development of a methodology partly based on informed assumptions or extrapolations, which cannot accurately represent the situation in every country. For example, the methodology for calculating an average animal weight from slaughter data, necessitates a conversion coefficient from carcass weight to live weight at time of slaughter. Presently, the European conversion coefficients were used for all the countries, but it is not currently known how well these apply to other countries that may have different slaughter practices, different breeds etc.

In the absence of global animal population data detailed by age and production, extrapolations were also calculated from European references. The extent to which these age class distributions of species apply to other countries is still undergoing a validation process.

The methodology for calculating biomass in several species is based on the mean standard weight of animals for breeding. An effort was undertaken to adapt these mean standard weights between regions using livestock units (13). A review of how well these standard weights depict the variability at a regional and national level has been initiated.

For cervids, camelids, and equines in some regions, data on breeding cycles were not collected at the time of reporting, nor was slaughter data. Therefore, this information was taken from literature where necessary, or extrapolated from regions where data was available (such as in the case of live weights of equines). The extent to which these literature and extrapolated weights and reproduction rates represent the true situation in any country is expected to vary.

Imported and exported animals are commonly subtracted and added, respectively, from animal populations when calculating animal biomass, as done in ESVAC and CIPARS. This is done so that only animals raised in the country, the time during which they would have been treated with antimicrobials, are considered. Currently, available data does not support incorporation of imported and exported animals. Their contribution to overall animal biomass was found to be relatively minor when calculated for certain countries where data was available.

In development of the current denominator methodology, it was decided at this time not to include companion animals in the calculation of animal biomass. Data on populations of cats and dogs are available in WAHIS, however, many countries do not report these figures, or report them inconsistently. Another consideration is the need to better understand whether reported cat and dog populations represent owned or stray animals, as this would affect the likelihood of their treatment with antimicrobials.

For the countries where cat and dog populations were available, it was seen that their contribution to overall biomass was minor (<1%). However, as some countries do include antimicrobials used in companion animals in their reported quantitative data, there is expected to be a small effect on results by excluding these species. As excluding them decreases this denominator, this effect, if any, would be a minor increase in antimicrobial quantities adjusted for animal biomass.

In the future, a goal would be to provide a separate analysis for antimicrobial agents used in companion animals, as more countries are able to report these population data, and distinguish antimicrobial quantities by animal group.

### Prospects

The OIE will continue working closely with its Member Countries to support them in calculating kilograms of active ingredients of antimicrobials. An automated system for this calculation (conversion of antimicrobial active ingredients in veterinary medicines into kilograms) will be developed over time to assist Member Countries in this effort. This automated system will particularly help Member Countries with the burden of manually calculating kilograms of active ingredients and avoid errors with these calculations.

The OIE will also continue to refine its methodology for the calculation of animal biomass, based on globally available data, and communication with its Member Countries through its regional offices.

An important next step in this process is collaboration with the OIE World Animal Health Information and Analysis Department (WAHIAD). In consultation with the OIE *ad hoc* Group on Antimicrobial Resistance, new species and animal sub-categories have been added to the OIE World Animal Health Information System (WAHIS) data collection guidelines. These new population sub-categories are now being implemented in WAHIS and will allow to refine the data on animal biomass over time.

OIE-WAHIS, the next generation of the WAHIS data collection interface, is currently in development and will incorporate further updates to the collection of global animal population data. In addition to more sub-categories representing detailed production data when Member Countries are able to supply it, the interface will also include free text boxes allowing for description of the reported data. OIE-WAHIS will also additionally support the reporting of data on average live weights and number of animals slaughtered in Member Countries.

Aside from collection of more detailed global animal population data, more work is needed to validate some of the conversion coefficients, breeding cycles and population distribution ratios used in the methodology, which were extrapolated from European data as necessary. Particularly, better understanding potential regional variation in carcass conversion coefficients (for estimating live weights) and annual multiplication rates of species living <1 year (i.e., “cycle factor”) are necessary within the current methodology to ensure its applicability on a global scale. The OIE is currently working with its Regional Offices to obtain better estimates on these variables across regions.

The third AMU report published by the OIE in 2019 clearly shows the significant commitment of OIE Member Countries to the development of data collection systems on antimicrobial agents intended for use in animals. The capacity to measure trends over time is progressing each year, and is critical to the international effort to promote the responsible and prudent use of antimicrobial agents in animals.

Simultaneously, as more precise data on animal populations becomes globally available, it is expected that the methodology for calculation of animal biomass will be further refined. With the concurrent development of quantitative data collection and calculation of animal biomass, this annual report will allow for increasingly effective comparisons on a global and regional scale.

## Data Availability

The raw data supporting the conclusions of this manuscript will be made available by the authors, without undue reservation, to any qualified researcher.

## Author Contributions

All authors listed have made a substantial, direct and intellectual contribution to the work, and approved it for publication.

### Conflict of Interest Statement

The authors declare that the research was conducted in the absence of any commercial or financial relationships that could be construed as a potential conflict of interest.

## Footnotes

^1^Point in time census data represents the number of living animals in a country at the time of survey.
